# The gynecologist and cancer in women

**DOI:** 10.61622/rbgo/2024rbgo92

**Published:** 2024-10-23

**Authors:** Iolanda Matias Gomes

**Affiliations:** 1 Universidade Federal de Pernambuco Recife PE Brazil Universidade Federal de Pernambuco, Recife, PE, Brazil.

Cervical cancer continues to claim an alarming number of victims around the world, especially among poor women. In Brazil, in 2022, an incidence of 16.3/100,000 women was recorded,^(1)^ with a projection for 2023 of 17,010 new cases, corresponding to a rate of 15.38/100,000, representing 7% of tumors in women.

In the Brazilian reality, where there is a great economic disparity by region, the incidence of this disease demonstrates its clear relationship with poverty and the consequent low access to health resources, with rates varying from 27.63/100,000 in Amazonas to 10.42/ 100,000 in Rio Grande do Sul.^(2)^ In November 2020, the World Health Organization released its plan to eliminate cervical cancer, with the expectation of a median reduction of 42% in the incidence of cervical cancer by 2045 and 97% by 2120, preventing a further 74 million new cases and saving 62 million lives in that period.^(3)^ In its strategy, 90% of girls up to 15 years of age must be vaccinated against HPV by 2030, 70% of women aged 35 to 45 properly screened, and 90% of women with the disease in the cervix must receive treatment.

Recommendations for eradicating cervical cancer are known and widely publicized. The Ministry of Health in Brazil announced, in March 2023, the incorporation of molecular testing for detecting Human Papillomavirus (HPV) into the public network. Part of this strategy includes increasing vaccination rates in adolescents aged 9 to 14.^(4)^

All of these measures aim to reduce new cases of the disease. It is known that the isolated impact of vaccination in reducing mortality in existing cases is only 0.1% by 2030.^(5)^ However, increasing screening and expanding cancer treatments would reduce mortality by 34.2% over the same period.

It is interesting to note that the one cited by the WHO^(6)^ to detect the disease early is associated with the disease symptoms already advanced:

- Unusual bleeding between periods, after menopause, or after sexual intercourse;
- Increased or foul-smelling vaginal discharge;
- Persistent pain in the back, legs or pelvis;
- Weight loss, fatigue, and loss of appetite;
- Vaginal discomfort;
- Swelling in the legs.

Increasing vaccination coverage and PCR testing for HPV are crucial measures, but a more realistic measure to reduce deaths from the ongoing disease is urgent. In addition to early diagnosis, we need to treat more, improve our approach to decision-making in gynecology outpatient clinics and colposcopy services, encourage and invest in the outpatient treatment of precursor lesions, and encourage "see and treat".

Brazilian gynecology accompanies women in all their stages of development and reproduction, menopause, and the pregnancy-puerperal cycle. But what about cancer in women? Notably at this point, they are referred to general surgery, breaking a cycle of trust and complicity that women have with their gynecologist.

There is, so to speak, a gap in care, where doctors with training in general/oncological surgery do not have the required skills and training in endocrine-gynecological knowledge for better decision-making. On the other hand, training in gynecology residencies does not prepare women for oncological surgeries.

Therefore, strengthening the care of women with cancer in conjunction with gynecology can be a promising path in the fight to reduce deaths among women with ongoing disease. Stimulating knowledge and developing skills for the surgical treatment of cases, in the various training centers for gynecologists, disseminating this commitment to women in Brazil is a path desired by many who are on the front line of the disease and see decades of removal of gynecology from cancer in women.
